# Probable Aerosol Transmission of SARS-CoV-2 through Floors and Walls of Quarantine Hotel, Taiwan, 2021

**DOI:** 10.3201/eid2812.220666

**Published:** 2022-12

**Authors:** Hsin-Yi Wei, Cheng-Ping Chang, Ming-Tsan Liu, Jung-Jung Mu, Yu-Ju Lin, Yu-Tung Dai, Chia-ping Su

**Affiliations:** Taiwan Centers for Disease Control, Ministry of Health and Welfare, Taipei, Taiwan (H.-Y. Wei, M.-T. Liu, J.-J. Mu, Y.-J. Lin, C.-p. Su);; Chang Jung Christian University, Tainan, Taiwan (C.-P. Chang, Y.-T. Dai)

**Keywords:** COVID-19, aerosol transmission, quarantine hotel, SARS-CoV-2, coronavirus disease, severe acute respiratory syndrome coronavirus 2, viruses, respiratory infections, vaccine-preventable diseases, Taiwan

## Abstract

We investigated a cluster of SARS-CoV-2 infections in a quarantine hotel in Taiwan in December 2021. The cluster involved 3 case patients who lived in nonadjacent rooms on different floors. They had no direct contact during their stay. By direct exploration of the space above the room ceilings, we found residual tunnels, wall defects, and truncated pipes between their rooms. We conducted a simplified tracer-gas experiment to assess the interconnection between rooms. Aerosol transmission through structural defects in floors and walls in this poorly ventilated hotel was the most likely route of virus transmission. This event demonstrates the high transmissibility of Omicron variants, even across rooms and floors, through structural defects. Our findings emphasize the importance of ventilation and integrity of building structure in quarantine facilities.

Transmission of SARS-CoV-2 through direct, person-to-person contact has been recognized since the early stages of the COVID-19 pandemic (*1*). However, mounting evidence suggests that the virus can be transmitted through the inhalation of virus-laden aerosols (*2*,*3*). Aerosols are small respiratory particles that can linger in the air and can disperse or travel over a distance of 2 meters under certain circumstances. Such transmission has been reported in restaurants, during a choir rehearsal, and at a bar (*4*–*6*). The World Health Organization and the US Centers for Disease Control and Prevention have officially acknowledged that aerosol transmission might occur in crowded indoor settings and poorly ventilated spaces (*7*,*8*).

Taiwan contains the COVID-19 pandemic mainly through tight quarantine measures for inbound travelers. In the weeks leading up to Lunar New Year of 2022 (February 1, 2022), there were strong demands for quarantine hotels because many overseas residents of Taiwan traveled back home. Consequently, many commercial hotels were adapted for use as quarantine hotels, although they were not designed for that purpose. Increased spread of the Omicron variant through aerosol transmission posed great risk for residents of these hotels, despite stringent quarantine measures.

The first outbreak of SARS-CoV-2 in a quarantine hotel in Taiwan was detected in December 2021. The outbreak affected 8 travelers and involved the Delta variant (*9*). Since then, 15 or more clusters of Omicron variant transmission have occurred within quarantine hotels; all clusters were confirmed by whole-genome sequencing (*10*,*11*). Previous reports in other countries likewise have revealed that aerosol transmission can happen across corridors (*12*–*14*) and floors (*15*–*18*) of quarantine hotels and apartments. Nonetheless, the relationship between disease transmission and building structure and ventilation remains largely unexplored.

On December 29, 2021, three cases of COVID-19 associated with a quarantine hotel in northern Taiwan were reported to the Taiwan Centers for Disease Control. The 3 patients stayed in nonadjacent rooms across floors and were diagnosed with COVID-19 during their quarantine period. The Taiwan Centers for Disease Control initiated an investigation to identify the infection source and possible transmission route and to recommend preventive measures.

## Methods

### Entry Screening and Quarantine Measures in Taiwan

Since December 2020, all travelers entering Taiwan must present a COVID-19 nucleic acid amplification testing report issued within 3 days before boarding a flight (*19*). Since July 2021, testing of a deep-throat saliva specimen for SARS-CoV-2 by reverse transcription PCR (RT-PCR) upon arrival at the airport also has been required (*20*). Because of the influx of inbound travelers before the Lunar New Year holiday in 2022, all travelers were also required to undergo a 7-day, 10-day, or 14-day quarantine at a quarantine hotel (*21*). Arriving travelers took a quarantine vehicle to a quarantine hotel or a group quarantine facility. Before the end of the quarantine, travelers were required to undergo another RT-PCR test. Those who tested positive for SARS-CoV-2 were immediately transferred to a designated hospital for isolation.

### Case Investigation and Contact Tracing

COVID-19 is a national notifiable disease in Taiwan. All RT-PCR–confirmed cases must be reported to public health sectors. We collected travel histories and laboratory findings for the 3 persons identified as COVID-19 case-patients in a quarantine hotel in northern Taiwan (case A, case B, case C). We defined close contacts as the guests who stayed on the same floor as the 3 case patients during December 22–29, 2021 (the period case B stayed in the hotel), because we believed those guests shared the same air with case B. For contact tracing, all staff workers and other guests in the building underwent a single RT-PCR test at the beginning of the investigation. The close contacts received an additional test at the end of quarantine, and the staff workers received tests every 3 days. We assumed case B to be the primary case because the patient travelled to Taiwan from New York, NY, USA, where active transmission of Omicron variants was occurring.

### Investigation of Noncontact Practice

Two senior infection control nurses interviewed the hotel staff and the hotel owner about the compliance criteria that determined the facility’s “noncontact” practice and inquired about specific preventative measures for preventing contact between the guests, such as food delivery, garbage collection, and respiratory sampling before the end of quarantine. The nurses also inspected personal protective equipment used at the hotel.

### Laboratory Investigation

We conducted whole genome sequencing on all specimens from the 3 case patients using Illumina COVIDSeq Test protocol on the iSeq 100 system (Illumina Inc., https://www.illumina.com) (*22*). The ARTIC v3 primers produced 98 amplicons and were designed to amplify SARS-CoV-2 virus-specific sequences. We processed the obtained viral sequences using DRAGEN COVID Lineage application version 3.5.5 on Illumina’s Basespace cloud analytical system and compared those with Nextclade software (https://clades.nextstrain.org). The median sequence read depth of the 3 samples was ≈4,000×, and ≥30× coverage achieved >98% sequence coverage. The genomic sequences of the 3 cases in this study were deposited in the GISAID database (https://www.gisaid.org; accession nos. EPI_ISL_13535670, EPI_ISL_13535983, EPI_ISL_13536113). We constructed phylogenetic trees using IQ-TREE software by using the maximum-likelihood method (*23*). We performed the phylogenetic analysis with sequences from the United States (1,988 viruses), Japan (79 viruses), and China (1 virus) from the same time frame.

### Environmental Investigation

We removed all quarantine guests from the building 2 days before the investigation. We checked the structural layout and ventilation system of the building by direct exploration. We observed the partition walls from the access opening. In the mezzanine above the ceiling—preserved for pipes, electrical wires, and air conditioners—we examined the integrity of the walls. We performed a simplified tracer-gas experiment to assess the interconnections between rooms. We detected total volatile organic compounds (TVOC) with an air quality monitor (INKBIRDPlus AK3, https://inkbird.aliexpress.com/) and used a CO_2_ sensor (IAQ-CALC Model 7515; TSI Incorporated, https://tsi.com) for validation of CO_2_ concentration. 

Ethanol (75%) from a spray can of hand sanitizer was used as an indicator. Ethanol is a volatile organic compound and is detectable by the air quality monitor. The volume of a single press of the spray releases ≈0.9 mL of ethanol. To simulate the real situation during the cluster, we turned on the bathroom exhaust fan and the air conditioner of each test room during the experiment. We released ethanol with 10–15 presses in room 510 (case B, suspected index patient) and took measurements in room 610 and room 611 (case A).

Using methods recommended by the World Health Organization (*24*), we took environmental virus swab specimens for RT-PCR tests to provide supplementary aerosol transmission findings. We took samples in the rooms of the 3 case patients and samples from public areas on the involved floors. We conducted environmental swab sampling on January 1, 2022, eight days after the checkout of case A and 3 days after the checkouts of case B and case C.

## Results

### Case Investigation and Contact Tracing

**Figure 1:**
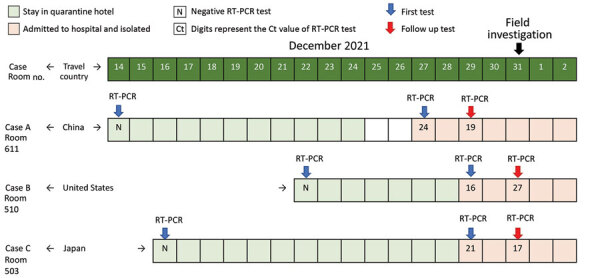
Timeline for Case A, Case B, and Case C in our investigation of aerosol transmission of the SARS-CoV-2 Omicron variant between separate, nonadjacent rooms in a quarantine hotel in Taipei City, Taiwan. Blue arrows indicate the results of the first RT-PCR testing, and red arrows indicate the results of the follow-up RT-PCR testing. The field investigation began on December 31, 2021.

The 3 case patients we studied all tested negative by RT-PCR for SARS-CoV-2 within 72 hours before arrival to Taiwan and by deep-throat saliva RT-PCR upon arrival at the airport (Figure 1). None had left their rooms at any point during the stay in the hotel. No other guest or staff member at the hotel had tested positive since the month prior to the start of the investigation.

Case A arrived in Taiwan from Shenzhen, China, on December 14, 2021, and was admitted to room 611 in the quarantine hotel. He had previously received 2 vaccine doses (Sinopharm, http://www.sinopharm.com); the second dose was given on August 11, 2021. He remained asymptomatic during the quarantine period.

Case B arrived in Taiwan from the United States on December 22, 2021, and was admitted to room 510 in the same quarantine hotel. He had previously received 2 vaccine doses (Pfizer-BioNTech., https://www.pfizer.com); the second dose was given on May 19, 2021. He claimed that he was asymptomatic until December 29, when he began to experience a sore throat.

Case C arrived from Japan on December 16, 2021, and was admitted to room 503. She had previously received 1 vaccine dose (Pfizer BioNTech), which was given on October 25, 2021. She remained asymptomatic during the quarantine period.

Because case A tested positive on December 28, the public health sector arranged RT-PCR tests the next day for hotel guests on the 5th and 6th floors to find the source. Cases B and C tested positive (Ct value for case B was 16; Ct value for case C was 21) on December 29. Both patients were transferred to the hospital for isolation.

All 8 members of the hotel staff had received 2 COVID-19 vaccinations and underwent enhanced surveillance RT-PCR testing every 3 days; each staff member had 5 negative test results after the transmission was recognized. The RT-PCR test results for the 70 other guests who stayed in the hotel during December 22–29, 2021, were all negative. The close contacts (14 guests on the 5th and 6th floors) were asked to quarantine for another 14 days, starting on December 29 (they were relocated to another quarantine hotel), and all tested negative before the end of the quarantine.

### Investigation of Noncontact Practice

The quarantine hotel we studied requires staff members to wear personal protection equipment when entering the guest zone (on any floor, except the staff zone on the 1st floor), including gloves, gown, goggles, cap, shoe covers, and a medical mask. There is a trash bin and a chair for meal placement in front of every room. The food delivery and garbage collection are carried out by noncontact means and occur at specific times of the day. Hotel guests are strictly prohibited from having contact with other guests and could exit their rooms only for RT-PCR testing. Guests are instructed to open the door to their rooms only when wearing a mask. They receive advanced notification by phone to take meals into the room and to proceed downstairs for RT-PCR testing by turns to avoid brief encounters with others. A bus designed for respiratory sampling stops at the hotel, as arranged by the public health sector.

### Laboratory investigation

**Figure 2:**
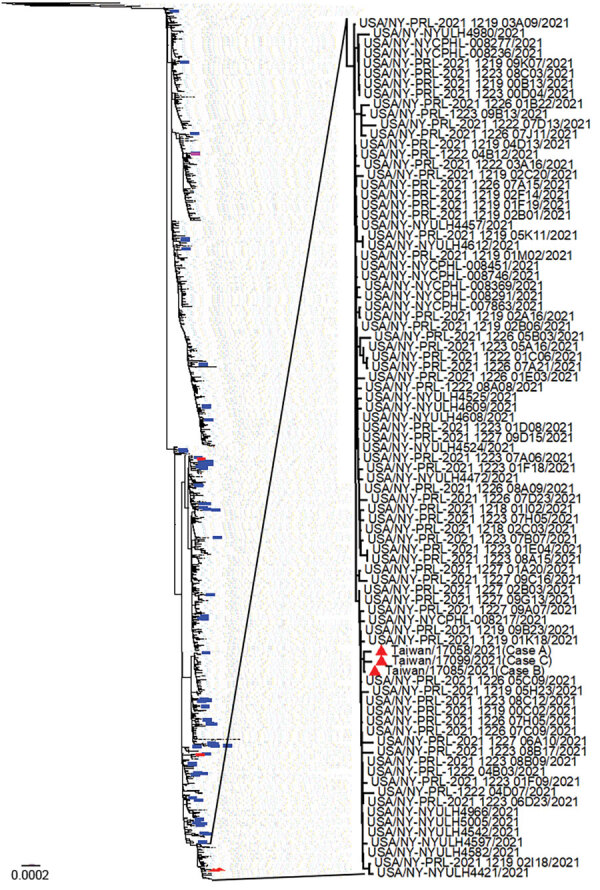
Whole-genome sequencing of SARS-CoV-2 from 3 case patients (indicated with red triangles) who contracted COVID-19 in a quarantine hotel in Taipei City, Taiwan, in December 2021 (GISAID with accession ID EPI_ISL_13535670, 13535983, 13536113). Phylogenetic trees were constructed using IQ-TREE software (http://www.iqtree.org/) by maximum likelihood. The phylogenetic analysis with sequences obtained from the United States (1988 viruses), Japan (79 viruses, blue lines), and China (1 virus, red line) sampled from the same timeframe revealed that sequences of the 3 case patients fell into a subclade close to one from the United States. Scale bar for enlarged tree section indicates substitutions per site.

We classified the virus sequences of specimens collected from the 3 case patients as the Omicron variant (BA.1.1 lineage). We conducted phylogenetic analysis with genomes from the United States, Japan, and China sampled from the same time frame, which revealed that the sequences of the 3 case patients fell into a subclade close to one from the United States (Figure 2). The sequences of the 3 case patients shared the same 3 unique single-nucleotide polymorphisms and differed by only 2 nucleotides from case A to case B (G17462A, C18877C) and case B to case C (C27874T).

### Environmental Investigation

#### Room and Corridor Air Ventilation

The quarantine hotel we studied is located in downtown Taipei City. The building consists of 7 floors and 24-hour central air conditioning powered by an air-cooled chiller unit on the roof; each room has an independent ceiling-mounted fan coil unit. The air in each room is 100% recirculated; no fresh air is brought in from outside of the building. The bathroom of each room has an exhaust fan that cofunctions with the ceiling lamp. The bathroom exhaust fan outlet terminates to the space above the ceiling and is not ducted outside of the building (Appendix Figure 1, Figure 2, panels A, B, F). Hence, the air extracted by the fan returns to the room and recirculates. There is no high-efficiency particulate air filter and no ventilation system connecting separate rooms and floors. The corridor, room 611 (case A), and room 510 (case B) have no external windows, but room 503 (case C) has a small window that remained shut most of the time. The detected CO_2_ concentration in room 510 (case B) was 562 ppm at the beginning of our investigation and 1,040 ppm in the presence of the investigation team (8 persons) after 10 minutes.

#### Investigation above the Ceiling

We found that partition walls above the ceiling, made of bricks and cement, had defects or gaps, mostly around pipes and electrical wires (Appendix Figure 2, panels B, C). Residual tunnels connected rooms 410 and 411, 510 and 511, 610 and 611, and 503 and 511; these tunnels might have been left when old pipes were removed (Appendix Figure 2, panels D, E, F). We also noted some abandoned and truncated pipes with protruding openings underneath the bottom of the bathtub in room 610 when we checked the ceiling of room 510 (Appendix Figure 2, panels G, H). We also noted some defects located in the partition wall between rooms 503 and 511 (Appendix Figure 2, panel I).

### The Tracer-Gas Experiment

Before the tracer-gas experiment, the concentrations of TVOC in the air were extremely low. After the ethanol (75%) was released from room 510, a detectable concentration of TVOC was observed in rooms 610 and 611 (Table).

**Table:** Concentrations of TVOC measured by detector in specific rooms of a quarantine hotel in Taiwan, December 2021*

Room no.	Baseline TVOC, mg/m^3^	Read time from release in room 510, min	TVOC, mg/m^3^)
611	0.013	6	0.163
610	0.011	13	0.134

*Ethanol (75%) was used as the indicator for detection of TVOC. TVOC, total volatile organic compounds.

### Environmental Sampling for RT-PCR

We collected a total of 20 specimens from the rooms of the 3 case patients and some public areas, including specimens from the exhaust fans, the air outlets of the fan coil units, the door handles on the corridor side, the spaces above the ceiling of each room, the stair handrail between the 5th and 6th floors, and the elevator buttons. Test results were all negative.

## Discussion

We conclude that an episode of SARS-CoV-2 Omicron variant transmission occurred in the quarantine hotel we studied in Taipei City, Taiwan. It was the first domestic cluster of Omicron variant in Taiwan. The case-patients had stayed in different rooms and even on different floors. They had no direct contact with each other during their stay. The Omicron variant is highly transmissible (*25*–*27*), so aerosol transmission was the most plausible route in this investigation of what we determined to be a poorly ventilated quarantine hotel. The special setting of this and other quarantine hotels (that is, facilities used to place persons in closed and separated rooms) provided a unique opportunity to see that the highly transmissible Omicron variant can cause infections between floors and through wall defects.

We deduce the primary patient was case B, who was from New York, NY, which at that time had emerging cases related to Omicron. Case A traveled from China and case C traveled from Japan, areas where there was no active transmission of Omicron variants (*28*–*30*). The results of serial RT-PCR tests of case B showed decreasing viral loads (Ct 16 to Ct 27), whereas the results for the other 2 case patients showed increasing viral loads, indicating case B was infected earlier than case A and case C. Phylogenetic analysis further supports our assumption of case B as the primary case, revealing a viral genome sequence from case B to be similar to one from the United States (Figure 2).

**Figure 3:**
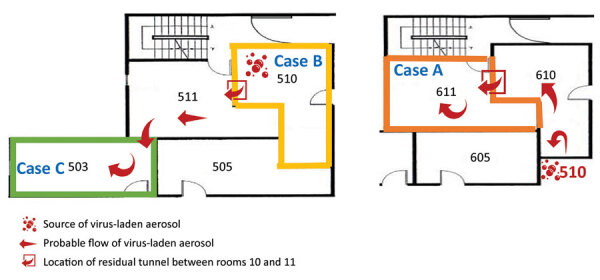
Simulation of probable air flow (arrows), implicating how virus-laden aerosol transported from the room of the primary case to the rooms of secondary cases in a quarantine hotel in Taipei City, Taiwan, December 2021. The bubbles symbol indicates the location of the source of transmission.

Room 611 (case A) and room 503 (case C) are not directly adjacent to room 510 (case B). We found truncated pipes above the ceiling in the room 510 bathroom, which might have connected to room 610, and a residual tunnel above the ceiling that might connect room 610 to room 611 (case A). A residual tunnel in the same location was also found in the middle of room 510 (case B) and room 511, and another tunnel connected room 511 and room 503 (case C). Because the air conditioners in each room are constantly pushing the air, the airflow carries the virus-laden aerosol. This aerosol might penetrate wall defects or go through old tunnels to reach other rooms (Figure 3), even nonadjacent rooms that appear to be independently isolated. When exhaust fans are not running at the same time (persons usually turn them on only when using the bathroom), it can result in pressure differences between rooms.

In addition to the obvious structural defects we discovered, tiny cracks in walls and ceilings also might have enabled air flow between rooms. To confirm that the wall defects and pipes interconnected the air in each room, we conducted a simplified tracer-gas experiment using methods similar to those used widely by petrochemical and automotive industries. The tracer-gas is released in small quantities into the main body of air to determine if leakage exists in a ventilation system. Previous studies used similar methods to simulate the process of potential transmission through air in 2 communities in China (*31*,*32*). Although those researchers chose sulfur hexafluoride, chloroform, and carbon tetrachloride as tracers, we chose ethanol as an index tracer because it is a detectable TVOC that is not harmful to humans and is easily obtained. Our goal was to demonstrate that 2 seemingly independent, closed rooms had hidden interconnections. Because of the lack of background TVOC measurements in rooms 503 and 505, the results were insufficient for comparison. However, we were able to deduce that the recirculated air transported through room 510 (case B) to room 503 (case C) based on observations in room 510 (case B) and room 611 (case A) during the experiment.

The hotel we studied had no fresh air supply to each room and no open window to the corridor on the guest floor, and the exhaust fan of each bathroom had no discharge tubes, indicating the air exhaled by the guest most likely stayed in the room. Because the concentration of CO_2_ could be affected by the number of persons present and their dwelling time, the high CO_2_ concentration we measured might reveal a poor ventilation rate but should be interpreted with caution. In the setting of underventilated indoor environments with recirculated air conditioning systems, the exhaled aerosol might remain suspended for a prolonged period and disperse across a long range (*2*,*33*,*34*). It is plausible, then, that a high concentration of virus-laden aerosol might have accumulated in a poorly ventilated room and might have been transported by the airflow across different rooms through the structural defects. Although the air flow might contain only small amounts of virus-laden particles, guests in quarantine stay in recirculated air for >1 week, making the risk of infection a logical possibility.

In Taiwan, at the time of the experiment, there were stringent requirements for hotels applying for quarantine status, such as daily environmental disinfection measures, no-contact service with adequate personal protection equipment, health surveillance, and infection control training programs for all staff members (*35*). However, there are not yet clear requirements relating to negative pressure capabilities, fresh air supply, full walls to the top, or air purifiers with high-efficiency particulate absorbing filters. As a reference, WHO has published a guide to improve indoor ventilation in the context of COVID-19 (*36*). Local governments in Taiwan have employed experts to carry out a comprehensive inspection of ventilation and air conditioning in all quarantine hotels (*37*,*38*). The inspection checklist noted some common structural problems: the partition wall of the guest room is not a solid wall to the ceiling, the gap of the pipe through the wall is not fully filled, the rooms have no dedicated supply of outdoor air, and the air from the bathroom leaks to other rooms or the corridor. Preliminary results of that inspection showed that around 25% of inspected hotels were ordered to address problems (*39*). We found almost all these problems in the single hotel we investigated; however, we note this as an extremely rare circumstance in which an unusual aerosol transmission occurred.

The first limitation of our investigation is that the simplified tracer-gas experiment used only 1 air quality monitor: a low-cost, non–research-grade instrument that was not calibrated before the experiment. In addition, TVOCs are ubiquitous in the indoor environment. Other sources of TVOCs in the rooms, including cleaning and beauty products, might interfere with readings. We should have compared the data of this tracer-gas experiment to the background instead of reading the absolute numbers. Second, ethanol is not an optimal surrogate for virus-laden aerosol, because an ethanol molecule is much smaller than a virus particle. The properties of vaporized organic solvent are different from exhaled droplets. The detection of a TVOC does not imply the possibility of virus transmission, but it did reveal structural defects. Third, instead of air sampling for the RT-PCR tests, we took only environmental swab samples to find evidence of virus-laden aerosols lingering in the rooms. Fourth, the capacity of the memory disc of the closed-circuit television video device in the public areas of the hotels was only 4 days, and we did not scrutinize it. Therefore, details such as frequency of door openings were not available.

Our findings support the possibility of SARS-CoV-2 aerosol transmission in a poorly ventilated quarantine hotel, which underscores the importance of ventilation and the integrity of building structure in selecting and approving quarantine facilities. To improve ventilation, a quarantine hotel should maintain a fresh air supply, and the exhaust gas from each quarantine unit must be properly collected and discharged. The building structure of a quarantine facility should be inspected to confirm that there is no gas exchange between units. The ethanol gas tracer test is a harmless, quick, and easy way to check for interconnections between rooms, a method that can be carried out by quarantine hotel personnel to confirm the integrity of rooms without requiring professional instruments or specialized techniques. To further reduce the risk for aerosol transmission in quarantine hotels, we recommend that the public health sector enhance surveillance during the quarantine period for inbound travelers, ensure good indoor ventilation, and promote public acknowledgment of aerosol transmission.

Appendix Additional information about probable aerosol transmission of SARS-CoV-2 through floors and walls in quarantine hotel, Taiwan, 2021.
