# Supplementary material for: Probable Aerosol Transmission of SARS-CoV-2 through Floors and Walls of Quarantine Hotel, Taiwan, 2021
Source: Emerg Infect Dis. 2022 Dec;28(12):2374–82. doi: 10.3201/eid2812.220666 (PMC9707602; doi:10.3201/eid2812.220666)
Supplement: Appendix — Additional information about probable aerosol transmission of SARS-CoV-2 through floors and walls in quarantine hotel, Taiwan, 2021. [file 22-0666-Techapp-s1.pdf]

Article DOI: https://doi.org/10.3201/eid2812.220666

Probable Aerosol Transmission of SARS-CoV-2 through Floors and Walls of Quarantine Hotel, Taiwan, 2021

Appendix

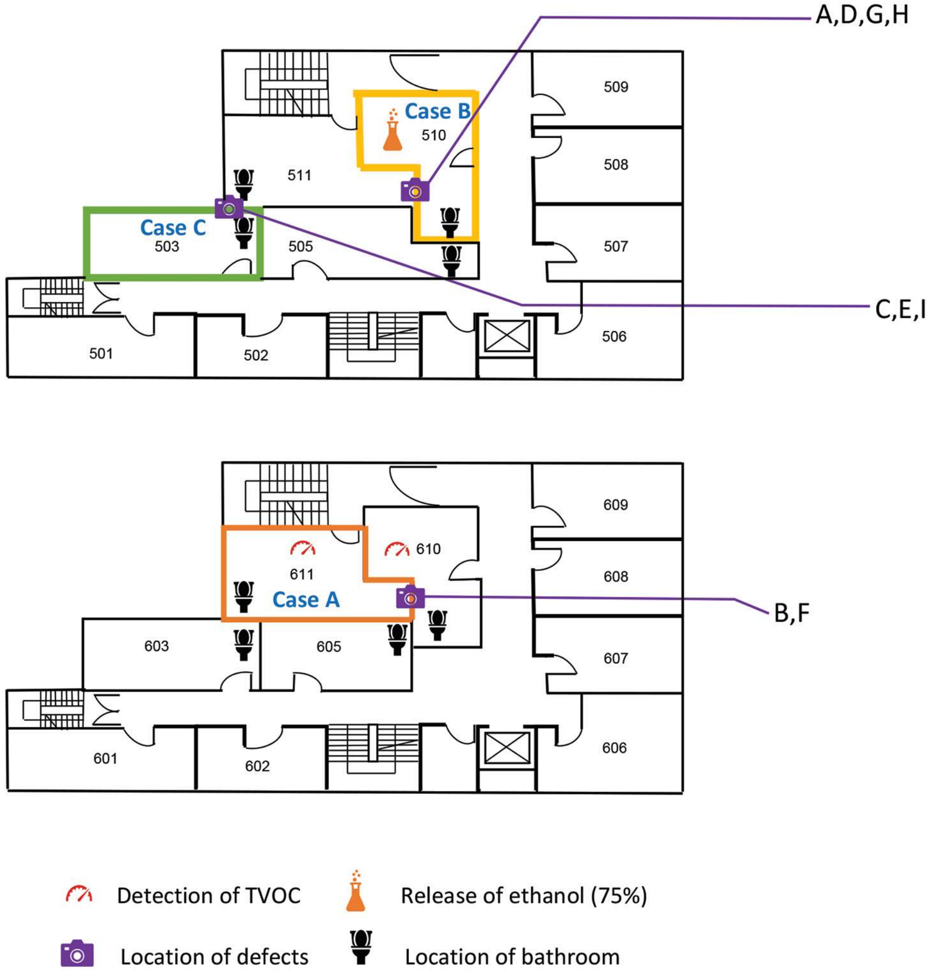

Appendix Figure 1. Layout of floors 5 and 6 of a quarantine hotel in Taipei City, Taiwan, December 2021, showing the relative locations of the tracer-gas experiments. Symbols indicate where investigators released ethanol (75%), location of the bathrooms, and where tracer gas was detected.

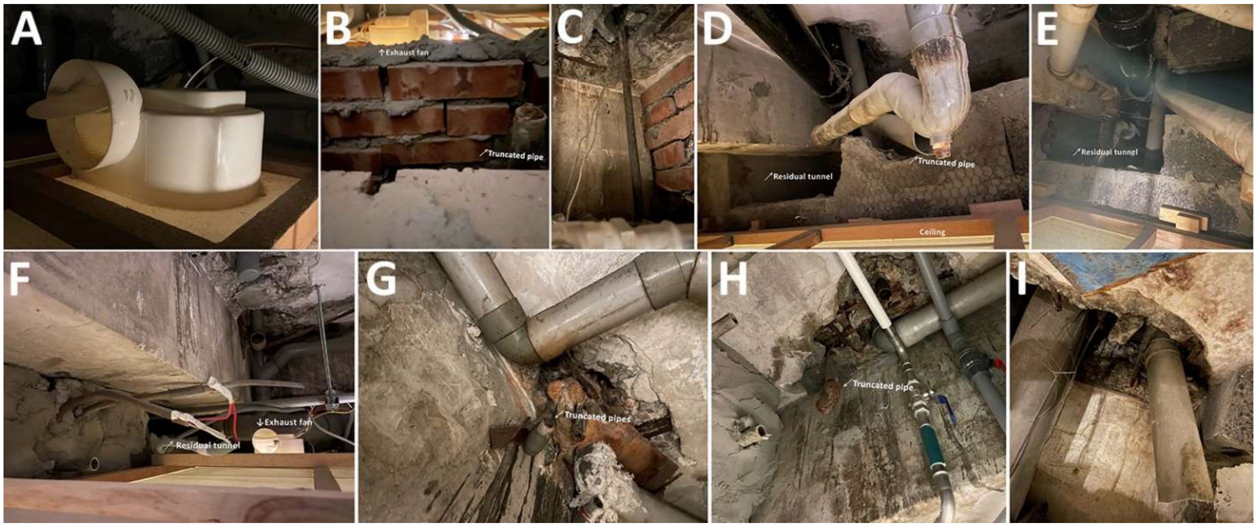

Appendix Figure 2. Photographs showing the tunnel between rooms and the structural defects between floors 5 and 6 of a quarantine hotel in Taipei City, Taiwan, December 2021. (A) Exhaust fan without collecting tube; (B) Bathroom, room 611. The wall above the ceiling was made up of bricks and cements in a rough way, truncated pipe from nowhere, and exhaust fan without a collecting tube; (C) Room 511. Wall defects with naked rebar; (D) Tunnel between room 510 and room 511 and truncated pipe (might connect to room 610); (E) Tunnel between room 503 and room 511; (F) Tunnel between room 610 and room 611; (G and H) Truncated pipes between room 510 and room 610; (I) Wall defects between room 503 and room 511.
